# Validation of radiological reduction criteria with intraoperative cone beam CT in unstable syndesmotic injuries

**DOI:** 10.1007/s00068-020-01299-z

**Published:** 2020-02-25

**Authors:** Sven Yves Vetter, Jeannie Euler, Nils Beisemann, Benedict Swartman, Holger Keil, Paul Alfred Grützner, Jochen Franke

**Affiliations:** MINTOS-Medical Imaging and Navigation in Trauma and Orthopaedic Surgery, BG Trauma Center Ludwigshafen At Heidelberg University Hospital, Ludwig-Guttmannstr. 13, 67071 Ludwigshafen, Germany

**Keywords:** Cone beam CT, Ankle mortise, Syndesmotic lesion, Syndesmosis

## Abstract

**Purpose:**

Acute unstable syndesmotic lesions are regularly treated with closed or open reduction and fixation with either a positioning screw or tight rope. Conventional fluoroscopy is limited to identify a malreduction of the ankle mortise. The aim of the study was to validate the reduction criteria of intraoperative cone beam CT in unstable syndesmotic injuries by analyzing the clinical outcome.

**Methods:**

Acute unstable syndesmotic injuries were treated with a positioning screw fixation, and the reduction in the ankle mortise was evaluated with intraoperative cone beam CT. The patients were grouped postoperatively according to the radiological reduction criteria in the intraoperative 3D images. The reduction criteria were unknown to the surgeons. Malreduction was assumed if one or more reduction criteria were not fulfilled.

**Results:**

Seventy-three of the 127 patients could be included in the study (follow-up rate 57.5%). For 41 patients (56.2%), a radiological optimal reduction was achieved (Group 1), and in 32 patients (43.8%) a radiological adverse reduction was found (Group 2). Group 1 scored significantly higher in the Olerud/Molander score (92.44 ± 10.73 vs. 65.47 ± 28.77) (*p* = 0.003), revealed a significantly higher range of motion (ROM) (53.44 vs. 24.17°) (*p* = 0.001) and a significantly reduced Kellgren/Lawrence osteoarthritis score (1.24 vs. 1.79) (*p* = 0.029). The linear regression analysis revealed a correlation for the two groups with the values scored in the Olerud/Molander score (*p* < 0.01).

**Conclusion:**

The reduction criteria in intraoperative cone beam CT applied to unstable syndesmotic injuries could be validated. Patients with an anatomic reduced acute unstable syndesmotic injury according to the criteria have a significantly better clinical outcome.

## Background

Traumas of the ankle are the most common injuries of the lower limb. Lesions of the syndesmotic complex in combination or without ankle fractures occur regularly [1–4].

Only a precise reduction and proper restoration of the anatomy can effectively prevent premature arthritis and painful limitation of the range of motion [3]. Operative treatment with a position screw using three or four cortices is widely applied in unstable syndesmotic injuries [5].

To achieve a precise anatomic reduction, several radiological parameters using conventional fluoroscopy with the standard views—AP (anterior–posterior), mortise, and lateral—are applied [6]. Nevertheless, multiple studies have revealed difficulties in anatomic reduction in the ankle mortise using conventional fluoroscopy [7–9]. Relevant differences of the tibiotalar, fibulotalar, and tibiofibular distances or angles of the fibula in the fibular notch can be concealed due to overprojection of anatomic structures and individual anatomic characteristics [10]. It has to be emphasized that the measured absolute values of distances of the ankle mortise have not been validated [11]. In unclear cases, a postoperative computed tomography is typically applied to detect malreduction [12]. However, intraoperative cone beam CT can be used to identify malreduction. Studies report an intraoperative revision rate due to intraoperative cone beam CT of up to 33% applying radiological reduction parameters [13, 14].

To the best of our knowledge, studies correlating a precise anatomic reduction using intraoperative cone beam CT to the clinical outcome do not exist.

The aim of the study was to validate the established reduction criteria in 3D imaging and to correlate these to the clinical outcome of patients with unstable syndesmotic injuries. The hypothesis of the study was that patients who fulfilled the established radiological reduction criteria had a better clinical outcome.

## Materials and methods

Adult patients with fractures of the upper ankle joint AO type 44 B and C with an additional lesion of the syndesmotic complex who underwent surgery between June 2002 and December 2010 with intraoperative 3D imaging were included in the study.

From June 2002 until December 2010, a total of 219 consecutive patients with lesions of the syndesmotic complex were treated operatively with stabilization of the syndesmosis using intraoperative 3D imaging. Ninety-two patients could not be included in the study due to the exclusion criteria (42%). Of the remaining 127 patients, 54 refused to attend or could not be contacted (43%). Therefore, 73 patients were included in the study (follow-up rate 58%).

Exclusion criteria are listed in Table 1.

**Table 1:** Exclusion criteria of the study

Injury of the ipsilateral extremity	Age < 18 years
Spinal cord injury	Former injuries of the ankle
Primary or secondary arthritis	Former infect
Revision surgery	Craniocerebral injury
Polytraumatization	Supervised person

In every case, preoperatively standardized X-rays in two planes (mortise and lateral) were obtained. An additional preoperative CT scan was not generated routinely but performed in cases of comminuted fractures in order to gain essential information. A comminuted fracture was defined if the fracture type of either malleolar presented with multiple fragments or a joint line impression.

After open reduction and internal fixation of the fibula, the medial malleolar fracture—if present—was addressed with open reduction and tension-band wiring or screw fixation according to the standards of the AO/OTA. In the case of a posterior malleolar fracture, an indirect osteosynthesis was conducted if the size of the fractured articular surface exceeded 20% or caused incongruency of the tibial notch in preoperative X-rays and in the intraoperative cone beam CT. In cases of comminuted fractures of the Volkmann fragment or the tubercle fragment, the anatomic reduction in the distal tibia was pursued to restore the fibular notch.

Subsequently, the stability of the syndesmotic complex was assessed by applying stress with the hook test described by Heim or Cotton test under fluoroscopy. If signs of instability—such as joint space widening, anterior–posterior fibular translation or fibular shortening—were present, a reduction in the distal fibula into the fibular notch restoring length, anterior–posterior translation and rotation was performed using a Weber clamp. The temporary fixation was performed using a 2.0 mm K-wire. After fluoroscopic examination, a 3.5-mm tricortical positioning screw was placed according to the technique of Heim [15]. In Maisonneuve fractures, a second screw was added.

With conventional fluoroscopy, the reduction was evaluated in the mortise and lateral view. If this proved positive under conventional fluoroscopy, a cone beam CT with SIREMOBILE Iso-C 3D (until March 15, 2005) or ARCADIS Orbic 3D (Siemens, Erlangen, Germany) was performed. With these motorized C-arms, 100 serial fluoroscopic images were obtained during a 190° orbital rotation, from which a 3D dataset was calculated with an edge length of 120 mm. Multiplanar reconstructions in the three standard planes (axial, sagittal, and coronal) were then created to assess the reduction quality and implant position. The fibula position in the tibial incisura was analyzed 10 mm proximal to the tibial joint line in the axial plane according to the recent literature [16]. The rotation of the fibula was assessed 6 mm distal the talar joint line [17].

**Fig. 1:**
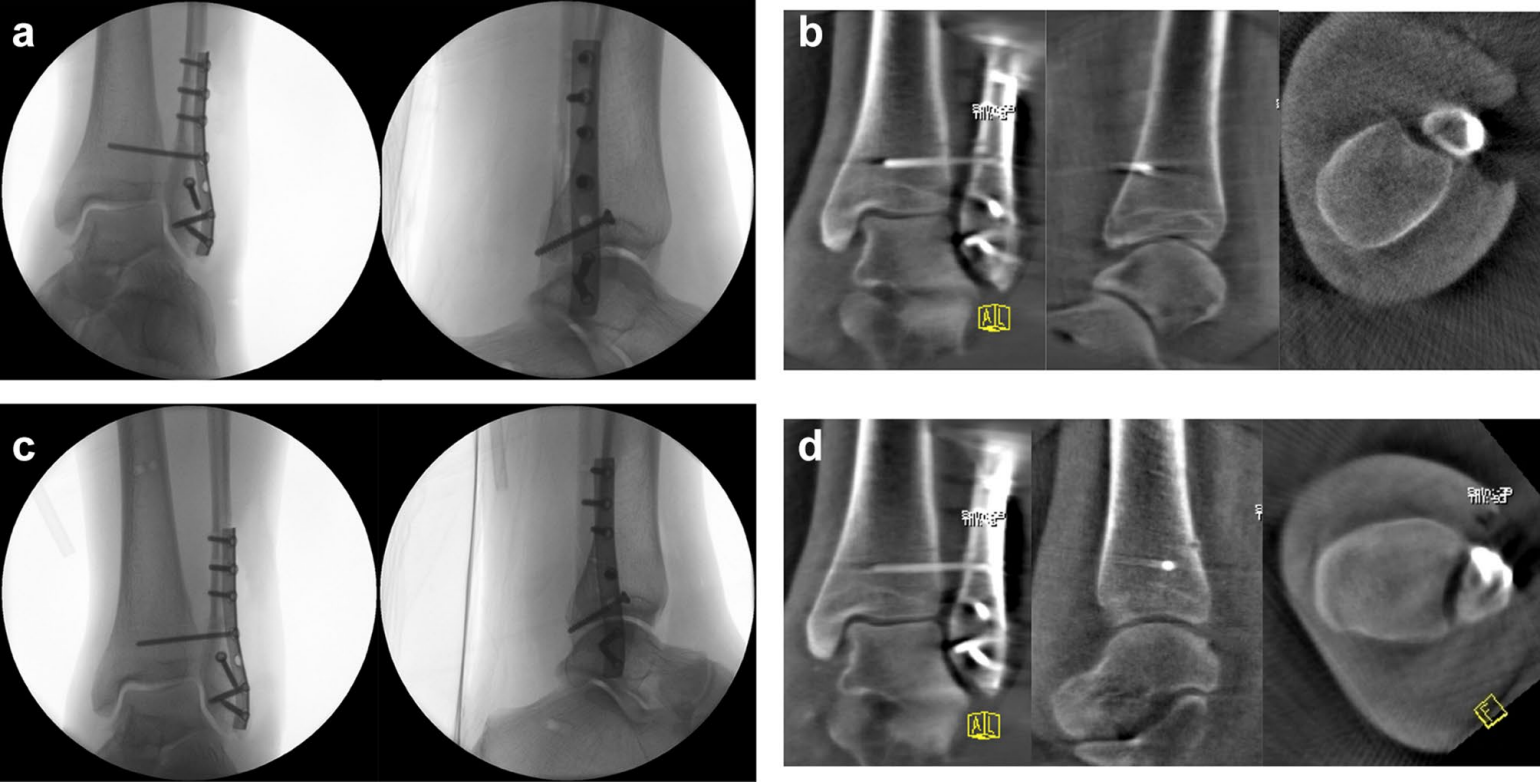
**a**–**d** Fluoroscopic images AP and lateral of the ankle after open reduction and internal fixation. In conventional fluoroscopy, anatomic reduction can be assumed (**a**). The cone beam CT images in the three standard planes demonstrate a posterior translation of the fibula (**b**). After correction, conventional fluoroscopy again reveals an anatomic reduction (**c**). The cone beam CT images now displays an anatomic reduction of the fibula in the fibular notch

If the images revealed a malreduction or implant misplacement, the correction was promptly conducted and again first of all controlled using conventional fluoroscopy. In the case of an adequate appearance of the correction, the result was verified again by cone beam CT (Fig. 1a–d).

A correct reduction in the ankle joint and implant placement in the 3D imaging were only defined if all of these parameters—related to the criteria proposed by Rammelt and Bartonicek—were fulfilled [18, 19]:

In the axial view:

1. Harmonious elliptical line between anterior margin of the fibula and the tibia in the tibial incisura 10 mm proximal to the tibial joint line (Fig. 2a).

**Fig. 2:**
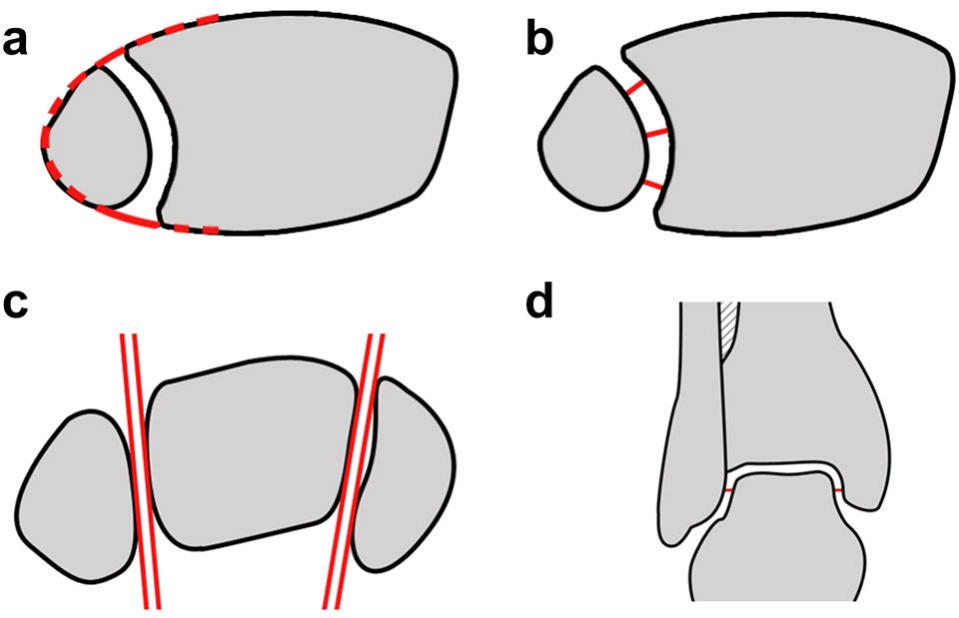
**a**–**d** Reduction parameters: Harmonious elliptical line between anterior margin of the fibula and the tibia in the tibial incisura 10 mm proximal to the tibial joint line (**a**). Symmetrical positioning of the fibula in the tibial incisura (**b**). Fibular rotation 6 mm distal to the talar joint line (**c**). Equal fibulotalar and tibiotalar clear space (**d**) in the coronal view
2. The fibula positioned in the tibial incisura correctly in relation to the topography and the width of the syndesmosis (Fig. 2b).
3. The correct rotation of the fibula reflected by congruent positioning of the malleoli in relation to the talus 6 mm distal the talar joint line (Fig. 2c).
4. Equal fibulotalar and tibiotalar clear space.

In the coronal and axial view:

1. Equal fibulotalar and tibiotalar clear space (Fig. 2d).
2. Anatomic length of the fibula.

In the coronal, sagittal and axial view:

1. No intraarticular implant placement.
2. Anatomical fracture reduction with steps and gaps of < 2 mm and without rotational deformity of the fibula.

The radiological criteria applied for this study were not conveyed to the surgeon prior to the operation. An experienced orthopedic surgeon, who was blinded, grouped the patients into two groups retrospectively. Patients were grouped in anatomic reduction (Group 1)—which fulfilled all radiological reduction criteria—or non-anatomic reduction (Group 2)—which failed to fulfill at least one criterion. Bimalleolar or trimalleolar fractures were evenly distributed in the two groups.

All patients were clinically and radiologically examined. The functional clinical outcome was assessed using the Olerud and Molander score and the range of motion. In addition, a multivariate analysis was generated for the Olerud and Molander score. The degree of osteoarthritis of the upper ankle joint was determined using the Kellgren/Lawrence score.

### Statistical methods

Statistics were performed using the software SPSS (Version 21.0.0.2, IBM Corporation, Armonk, New York, USA). A *t* test for paired samples was used to evaluate the results of the Olerud and Molander score, the Chi-square test was applied for the Kellgren Lawrence score, and the Mann–Whitney *U* test for the range of motion. A multivariate analysis was generated for the Olerud and Molander score. A two-sided significance at a significance level of α < 0.05 was set. If the result was less than α, a significant difference was assumed. A regression analysis for the Olerud/Molander score was conducted with age, gender, accident type, fracture type, and number of risk factors.

## Results

The demographic data age, gender, body mass index (BMI), and fracture classification according to the Orthopedic Trauma Association (OTA) classification are provided in Table 2.

**Table 2:** Demographic data age, gender, BMI, and fracture classification according to the OTA classification

Age (years; SD)	43.84 ± 14.12
Gender (%)	
Female	34
Male	66
BMI (kg/m^2^; SD)	28.47 ± 4.78
OTA Classification	
44-B	11
44-C	57
Isolated syndesmotic lesion	5

The mean follow-up time was 59 ± 28 months (min. 24 months, max. 125 months). The transfixation of the syndesmotic complex was performed with one tricortical screw in 53 patients and with two tricortical screws in 20 patients. Distal fibula fracture occurred in 67 patients and was treated with open reduction and internal plate fixation. Of the 27 fractures of the malleolus medialis, 11 were treated with tension screw and 16 with tension-band wiring. Fractures of the posterior malleolar (in 21 patients) were either treated with indirect screw fixation (7 patients, 33%) or treated conservatively (14 patients, 67%). An open reduction in the syndesmosis was performed in 59 cases (81%), and a closed reduction in 14 cases (19%).

Of the 73 patients included in the study, a radiological optimal reduction was achieved in 41 patients (56%) (Group 1) and a radiological adverse reduction was found in 32 patients (44%) (Group 2). Data derived from the 3D scan about the nature of the malposition of the distal end of the fibula after syndesmotic screw insertions were available for all 32 of the 73 patients. Four of the 32 patients (13%) showed isolated anterior displacement of the fibula in the fibular notch, and 3 (9%) showed isolated posterior subluxation of the fibula. The ankle mortise was persistently wide in three patients (9%). Isolated internal rotation of the lateral malleolus was present in ten patients (31%), and isolated external rotation was seen in five patients (16%). The combination of anterior displacement and internal rotation was found in two patients (6%), whereas anterior displacement and external rotation were not found. Combinations of a rotational component and posterior displacement were observed in two cases (6%).

The results revealed a significantly lower score of Group 1 (1.24) in the osteoarthritis Kellgren/Lawrence score compared to Group 2 (1.79) (*p* = 0.029). In ten patients, X-ray examinations could not be obtained due to pregnancy or missing medical indication.

The limitation of the range of motion (ROM) also differed significantly between the two groups (*p* = 0.00). ROM decreased by 24° in Group 2 in comparison with Group 1 (53°).

Group 1 reached a mean of 92.44 ± 10.73 (range: min. 50, max. 100) and Group 2 a mean of 65.47 ± 28.77 (range min. 5, max. 100) in the Olerud/Molander score (*p* = 0.00). In the linear regression analysis, only the gender (*p* = 0.003) and the group membership (*p* = 0.000) were correlated, as shown in Table 3.

**Table 3:** Multivariate regression analysis

Coefficients^a^					
Model	Non-standardized coefficients		Standardized coefficients	*t*	Sig.
	*B*	Std. error	Beta		
Binary coded variables^b^					
Constant	97.109	14.593		6.654	0.000
Group	− 22.281	4.912	− 0.454	− 4.536	0.000
Gender	15.566	5.347	0.303	2.911	0.005
Insurance	− 7.325	4.965	− 0.141	− 1.475	0.145
Smoking	− 1.926	4.906	− 0.038	− 0.393	0.696
Alcohol	0.511	5.221	0.010	0.098	0.922
Metric coded variables^c^					
Constant	90.196	19.630		4.595	0.000
BMI	0.172	0.606	0.033	0.283	0.778
Age	− 0.319	0.202	− 0.187	− 1.582	0.118

^a^Dependent variable: Olerud and Molander score

^b^Binary coded variables of the result in terms of the Olerud and Molander score

^c^Metric coded variables of the result in terms of the Olerud and Molander score

## Discussion

The aim of this retrospective single-center cohort study was to validate the radiological reduction criteria in unstable syndesmotic injuries with the clinical outcome. To the best of our knowledge, studies dealing with the correlation of intraoperative 3D imaging criteria and the clinical outcome of patients with acute lesions of the syndesmotic complex have not yet been published. The hypothesis of the study was that patients who fulfilled the established radiological reduction criteria had a better clinical outcome.

It is well known that the anatomic reduction and reconstitution of the syndesmotic complex and the ankle mortise are a crucial predictor of the postoperative clinical outcome after syndesmotic injury [3, 20].

Evaluation of the ankle joint in conventional X-ray has been established for years [21–23]. However, several recent publications have demonstrated several limitations of conventional fluoroscopy in analyzing the reduction in the ankle joint [13, 14]. A malreduction of the ankle mortise in > 20% was detected when conventional fluoroscopy was used intraoperatively for unstable syndesmotic injuries. Only Summers et al. revealed different results for the intraoperative use of conventional fluoroscopy [6]. The group published a rate of 5.5% (1 out of 18 patients) malreduced ankles for their approach that used conventional fluoroscopy of the uninjured side as a blueprint. However, it needs to be emphasized that the non-validated results included a small number of patients and that the procedure has not been implemented in any other study to support their hypothesis. Besides X-rays, computed tomography is the gold standard for identifying syndesmotic diastasis postoperatively [24]. Revision surgery is therefore regularly performed in the case of non-anatomic reduction, to avoid the adverse effects of malreduced ankles [25, 26].

Sagi et al. reported that 27 out of 68 (39%) operatively treated patients with an unstable syndesmotic injury were malreduced in a 2-year postoperative CT follow-up [25]. These patients had a significantly depleted functional outcome in the Short Form Musculoskeletal Assessment (27 ± 23.3 vs. 12 ± 10.6) and the Olerud/Molander (46.3 ± 28.5 vs 72.7 ± 22.5) questionnaires. Similar results could be seen in the present study. The functional outcome in the Olerud/Molander score was significantly lowered (65.47 vs. 92.44) in patients without anatomic reduction in the intraoperative 3D images.

Wikerøy et al. compared the operated and the non-operated ankle after syndesmotic injury using computed tomography in a mean follow-up of 8.4 years [26]. In 10 out of 48 (20.8%) cases, the width of the syndesmosis 10 mm proximal from the ankle joint line differed significantly (≥ 1.5 mm) between both sides. The clinical result measured with the modified American Orthopaedic Foot and Ankle Society ankle hind foot score was lowered in cases of malreduction with widening of the syndesmosis ≥ 1.5 mm. The authors did not differentiate between the types of malreduction (rotational, translational, etc.). Our results support these findings and accentuate a depleted clinical outcome in the case of a non-anatomic reduction in the syndesmotic complex.

Vasarhelyi et al. describe methods to determine fibula torsion and detect pathologies after operative treatment of unstable syndesmotic injuries by analyzing axial planes of the postoperative CT [27]. Three different locations to measure fibula torsion were defined, and the results were compared to the clinical outcome. A bilateral comparison was conducted. The measurement method showing the highest correlation between torsion deformity and clinical outcome analyzed the torsional angles of the fibula at the proximal and distal tibiofibular joint. The authors describe a high anatomic variability and conclude that fibular torsion asymmetry has a significant clinical relevance. Our data support these findings that non-anatomical reduction in the fibula into the tibiofibular notch is associated with a minor clinical result. A location to measure fibular torsion distal to the talar joint line was not considered. The publication fails to provide detailed information of the exact position of the axial planes of the CT, which means these findings are challenging to reproduce.

A solely X-ray-based study from Chissel and Jones demonstrated a decrease in functional outcome if widening of the syndesmosis was detected ≥ 1.5 mm [28]. Our data support these results and identify a significant reduction in the ROM of the ankle joint in malreduced ankles. In addition, our results show that patients with an anatomic reduced ankle mortise scored significantly higher in the Olerud/Molander score and the linear regression analysis showed significant differences in the group membership.

The study of Bartonicek et al. underline the importance of imaging in the treatment of unstable syndesmotic injuries [18]. In their study, the authors emphasize on the axial views of the ankle mortise to analyze fibular reduction into the notch. This is in accordance with our findings that intraoperative cone beam CT provides important additional information to achieve anatomical reduction in the ankle mortise.

One limitation of the study is the use of two different 3D mobile C-arms and the diverse image quality of the data obtained due to artifacts evoked by the implants used, bone quality, soft tissue swelling, and patients´ body mass index (BMI).

The mobile C-arms exhibited a minimal difference in image quality and scanning time, but this did not seem to affect the examination. Image artifacts did not significantly influence the results either. A more critical limitation is the radiological criteria applied for correct syndesmotic reduction. Potential CT criteria for an anatomic reduction in the ankle were set by Ebraheim et al. who conducted a CT-based cadaveric study [8, 29]. However, a simple endorsement of these criteria with respect to the intraoperative 3D imaging is crucial, as these criteria consider neither rotational differences of the distal fibula in the tibiofibular incisura nor the situation of unstable ankle injuries. However, studies have shown that interindividual anatomic variations make it even more difficult to rely on fixed parameters [8, 24, 29, 30]. Regular scanning of the contralateral uninjured side to obtain reliable data for reduction is critical due to an increased radiation exposure.

A pre- and postoperative CT scan was not performed routinely. Therefore, important information demonstrating additional pathologies of the ankle mortise may have been missed. The conventional CT offered superior image quality compared to the intraoperative cone beam CT. Nonetheless, intraoperative cone beam CT provided sufficient information to evaluate fracture reduction, ankle mortise, and positioning of the fibula into the notch. These findings are supported by several studies using cone beam CT to analyze the ankle mortise [31–34].

Another limitation of the study is the follow-up rate of 58%, which is most likely influenced by the large time interval of the study. The first operations were performed in 2002, and several patients could no longer be contacted.

In addition, it has to be taken into account that comminuted fractures of the Volkman or tubercle fragment may have led to an incongruency of the fibular notch which has influenced the evaluation of the reduction.

## Conclusion

In summary, the established radiological reduction criteria play a pivotal role, and if fulfilled significantly influence the postoperative clinical outcome of the patient. Due to these data, an anatomic reduction in the ankle mortise should be the main goal of the operational procedure. Cone beam CT can be used to analyze the reduction intraoperatively and, if necessary, correct this without the need for additional revision surgery. If an intraoperative cone beam CT is not available, then postoperative computed tomography is recommended for the treatment of unstable syndesmotic injuries.

## Data Availability

The datasets used and/or analyzed during the current study are available from the corresponding author on reasonable request.
